# Dosimetric evaluation of photons versus protons in postmastectomy planning for ultrahypofractionated breast radiotherapy

**DOI:** 10.1186/s13014-022-01992-w

**Published:** 2022-01-29

**Authors:** Puntiwa Oonsiri, Chonnipa Nantavithya, Chawalit Lertbutsayanukul, Thanaporn Sarsitthithum, Mananchaya Vimolnoch, Tanawat Tawonwong, Kitwadee Saksornchai

**Affiliations:** 1 Division of Radiation Oncology, Department of Radiology, King Chulalongkorn Memorial Hospital, The Thai Red Cross Society, Bangkok, Thailand; 2 Division of Radiation Oncology, Department of Radiology, Faculty of Medicine, Chulalongkorn University, Bangkok, Thailand

**Keywords:** Proton therapy, Ultrahypofractionation, Postmastectomy, Breast irradiation

## Abstract

**Background:**

Ultrahypofractionation can shorten the irradiation period. This study is the first dosimetric investigation comparing ultrahypofractionation using volumetric arc radiation therapy (VMAT) and intensity-modulated proton radiation therapy (IMPT) techniques in postmastectomy treatment planning.

**Materials and methods:**

Twenty postmastectomy patients (10-left and 10-right sided) were replanned with both VMAT and IMPT techniques. There were four scenarios: left chest wall, left chest wall including regional nodes, right chest wall, and right chest wall including regional nodes. The prescribed dose was 26 Gy(RBE) in 5 fractions. For VMAT, a 1-cm bolus was added for 2 in 5 fractions. For IMPT, robust optimization was performed on the CTV structure with a 3-mm setup uncertainty and a 3.5% range uncertainty. This study aimed to compare the dosimetric parameters of the PTV, ipsilateral lung, contralateral lung, heart, skin, esophageal, and thyroid doses.

**Results:**

The PTV-D95 was kept above 24.7 Gy(RBE) in both VMAT and IMPT plans. The ipsilateral lung mean dose of the IMPT plans was comparable to that of the VMAT plans. In three of four scenarios, the V5 of the ipsilateral lung in IMPT plans was lower than in VMAT plans. The Dmean and V5 of heart dose were reduced by a factor of 4 in the IMPT plans of the left side. For the right side, the Dmean of the heart was less than 1 Gy(RBE) for IMPT, while the VMAT delivered approximately 3 Gy(RBE). The IMPT plans showed a significantly higher skin dose owing to the lack of a skin-sparing effect in the proton beam. The IMPT plans provided lower esophageal and thyroid mean dose.

**Conclusion:**

Despite the higher skin dose with the proton plan, IMPT significantly reduced the dose to adjacent organs at risk, which might translate into the reduction of late toxicities when compared with the photon plan.

## Introduction

There are many techniques and schedule schemes of treatment for breast irradiation [1]. The ultrahypofractionation is the recent new schedule that can shorten the irradiation period. This study is the first dosimetric investigation comparing ultrahypofractionation using volumetric arc radiation therapy (VMAT) and intensity-modulated proton radiation therapy (IMPT) techniques in postmastectomy treatment planning.

Adjuvant radiotherapy plays a significant role in local or locoregional breast cancer treatment. The omission of breast irradiation significantly increases the recurrence risk [2–5]. Routine breast irradiation using three-dimensional conformal radiotherapy (3D-CRT) is associated with long-term toxicity in organs at risk (OARs), such as the heart, the lungs, and the contralateral breast, especially when regional node irradiation is required [6]. By using advanced techniques, such as intensity modulation radiotherapy (IMRT) or volumetric arc radiation therapy (VMAT), a high radiation dose could be delivered to the planning target volume (PTV) with high conformity. Although the high-dose regions of the lungs and heart are spared using these methods [7], but a higher volume of the adjacent OARs receives a low dose radiation, which can cause long term toxicities and secondary cancer [8]. A previous study used deep inspiration breath hold to reduce the radiation dose to the heart for left-side breast irradiation; however, a low dose to the remaining organs could not be eliminated [9].

With proton beam therapy (PBT), a well-known Bragg peak characteristic could potentially reduce the dose to OARs beyond the target almost completely. It is challenging to evaluate potential benefits for breast cancer, especially in chest wall treatment, which has a shallow depth. However, theoretically, proton therapy could reduce radiation doses to the lungs and heart by taking advantage of the rapid dose fall-off of the proton energy after the Bragg peak.

The conventional schedule for chest wall irradiation is 45–50 Gy in 25 fractions over 5 weeks. In the past decade, several trials investigated hypofractionation chest wall irradiation, defined as 43.5 Gy in 15 fractions for 3 weeks. Hypofractionation was noninferior to the standard scheme and with comparable toxicities to conventional fractionation among postmastectomy patients [10, 11]. A new study regimen of 26–27 Gy in 5 fractions for 1 week has been introduced in breast irradiation in the name of FAST-Forward [12]. Since the COVID-19 pandemic, ultrahypofractionated treatment has emerged as an option to reduce radiotherapy courses in the United Kingdom [13]. After the publication of the FAST-Forward study, the results showed that the acute skin reactions observed in FAST-Forward were mild and that the late normal tissue was noninferior to the results achieved using 43.5 Gy in 15 fractions [12, 14]. Breast irradiation with ultrahypofractionation has been applied in our department to reduce costs, and it may provide psychosocial benefits for patients [15, 16]. To our knowledge, there is no consensus on dose-volume constraints for PBT in ultrahypofractionation schemes. This is the dosimetric study to compare VMAT and intensity modulated proton radiation therapy (IMPT) in postmastectomy cancer patients.

## Materials and methods

This study was approved by the Institutional Review Board (IRB: 017/64). Twenty postmastectomy patients (10-left and 10-right sided) who received breast irradiation at King Chulalongkorn Memorial Hospital were included in this study. The computed tomography (CT) datasets from their treatment were replanned using ultrahypofractionation with both VMAT and IMPT techniques. All patients were in the supine position and had a straight face with both arms over the head. The patient was immobilized using Vac-Lok (CIVCO Medical Solution, Iowa, USA) and knee support (CIVCO Radiotherapy, Iowa, USA). The image dataset was acquired by Siemens SOMATOM Definition AS 64-slice (Siemens, Erlangan, Germany) CT simulation with a 3-mm slice thickness. The scan range, from C2 to L2, included all breast tissue [17], while wires marked the area of the chest wall during simulation.

The clinical target volume (CTV) included the chest wall and regional nodes (i.e., supraclavicular, axillary (level I–III) and internal mammary nodes), and organs at risk were contoured and reviewed by at least 3 radiation oncologists who specialized in breast irradiation. The CTVs of the breast and regional lymph nodes were contoured based on the Radiotherapy Comparative Effectiveness atlas (RADCOMP) [18].

The PTV was a 5-mm expansion from the CTV but extracted from the skin surface for 5 mm and from the lung/chest wall interface for 3 mm. All organs at risk, including the ipsilateral and contralateral lungs and the heart, thyroid, and esophagus, were contoured. The skin, which was a layer of 5 mm inward from the body, was also created.

**Fig. 1:**
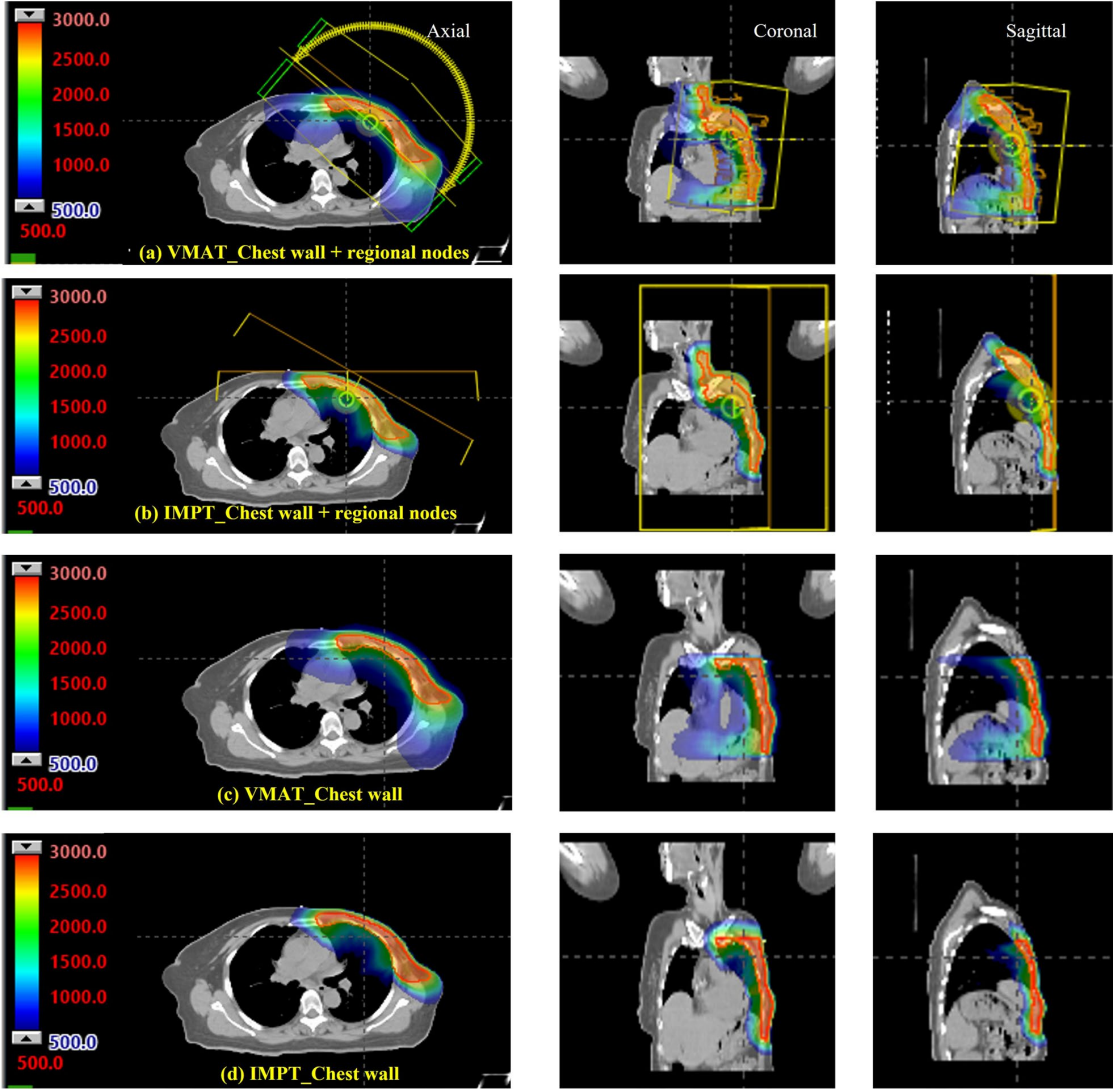
Comparison of color wash isodose distribution for the left chest wall with and without regional lymph nodes for VMAT versus IMPT planning: **a** VMAT chest wall + regional nodes; **b** IMPT chest wall + regional nodes; **c** VMAT chest wall; **d** IMPT chest wall

The wires placed during the simulation were overridden by a CT number of HU = − 1000 to approximate the air density. The VMAT and IMPT plans were generated by the Eclipse treatment planning system version 15.6 (Varian Medical System. Inc., Palo Alto, CA). Each patient had 2 PTVs: the PTV chest wall and the PTV chest wall including regional nodes. Therefore, four scenarios were created: left chest wall, left chest wall including regional nodes, right chest wall, and right chest wall including regional nodes. The prescribed dose was an ultrahypofractionated dose of 26 Gy (RBE) in 5 fractions, employing a generic relative biological effectiveness (RBE) value of 1.1 for proton plans [19]. The VMAT optimization was planned with 6 MV photon beams. A 1-cm bolus was added for 2 in 5 fractions. The VMAT plans consisted of 4 arcs at gantry angles 240°–50° and 135°–310° for the right and left breasts, respectively. The collimator was rotated 90° for 2 arcs, splitting the jaw to cover the upper part and lower part of the PTV. This was done to increase the dose coverage in the PTV due to the limitation of the maximum leaf span of the MLCs in Varian linear accelerators of the X-direction jaw of only 16 cm [17]. The IMPT plans were created with two fields: an anteroposterior (AP) field (0°) and an anterior oblique field with a gantry angle ranging from ± 30° to 45° depending on the beam direction to be as enface as possible to the chest wall. An example of a field arrangement is demonstrated in Fig. 1. A 5-cm range shifter was used to modify the beam dosimetric cover at all depths. This is the typical way to treat target at shallower depth than the minimum range of proton energy in proton treatment [20]. No bolus was applied to IMPT plans due to the reproducibility on bolus placement are seriously concern in proton treatment [20]. Additional targets were 0.5 cm for the proximal and distal margins and 1 cm for the lateral margin. Robust optimization was performed on the CTV structure with a 3-mm setup uncertainty and a 3.5% range uncertainty. The plans were normalized so that PTV received ≥ 95% of the prescription dose (95% of the prescribed dose was 24.7 Gy(RBE)) [6]. Dmean, V5, V10 and V20 were investigated in the heart and in the ipsilateral and contralateral lungs. The thyroid, esophagus, and skin were evaluated in Dmax and Dmean. The Dmax was 1% of the evaluation volume due to greater robustness than single point consideration [6]. STATA version 1.5.1 (Stata Corp LLL, Texas, USA) was used to perform statistical analysis. The paired t-test was used when data were normally distributed. The Wilcoxon signed- rank test was also used to analyze the difference in dose volume histogram between the VMAT and IMPT techniques in each scenario when the data did not follow a normal distribution. A *P* value < 0.05 was considered statistically significant.

## Results

**Fig. 2:**
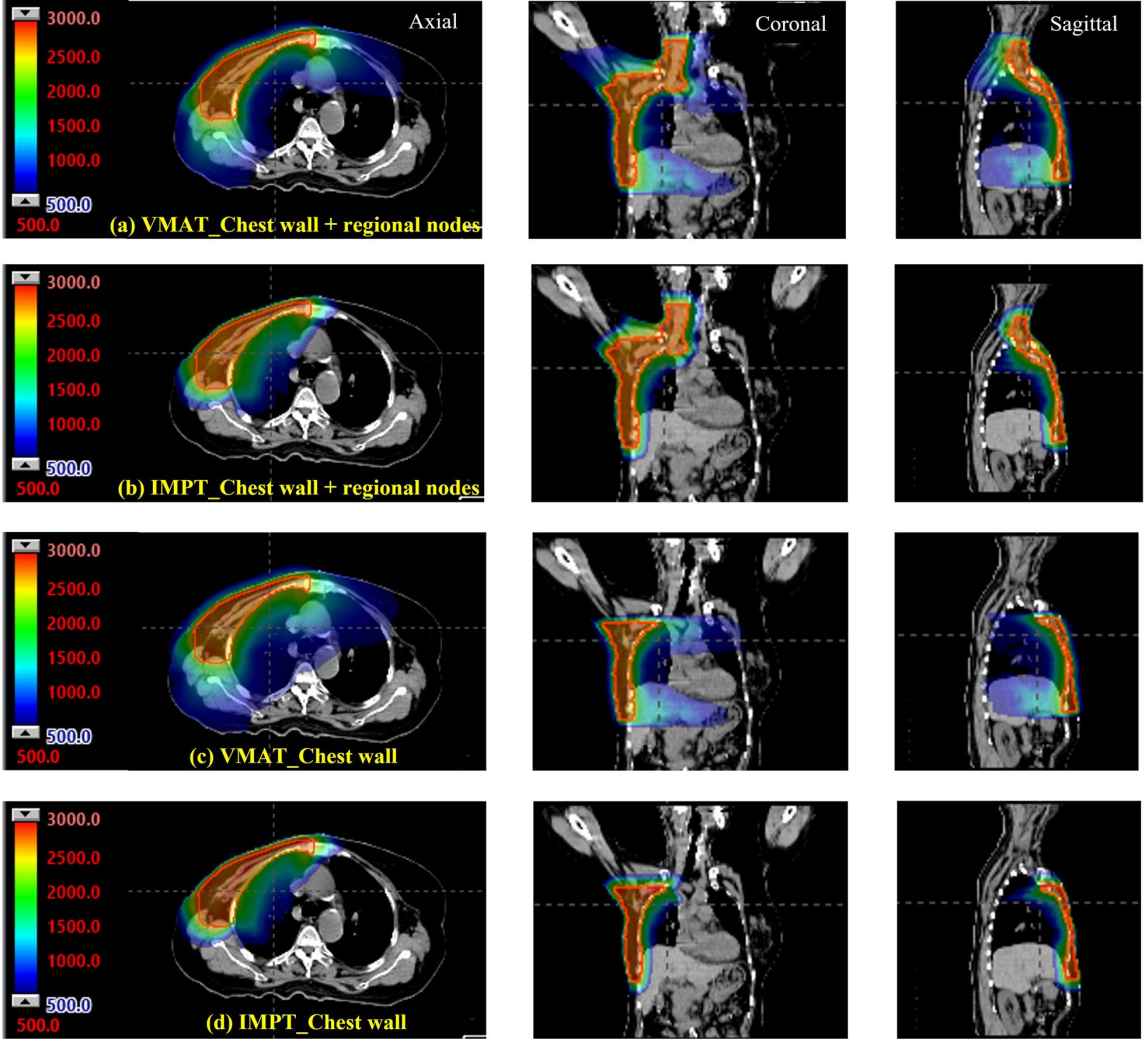
Comparison of color wash isodose distribution for the right chest wall with and without regional lymph nodes for VMAT versus IMPT planning: **a** VMAT chest wall + regional nodes; **b** IMPT chest wall + regional nodes; **c** VMAT chest wall; **d** IMPT chest wall

The results of the dosimetric comparison for the VMAT versus the IMPT plans are shown in Table 1. In both techniques, the approximate dose for D90 of PTV volume was higher than 25 Gy(RBE) on both sides of the chest wall. VMAT achieved more dose homogeneity and had a lower maximum dose than IMPT. Examples of dose distributions comparing IMPT and VMAT on the left and right sides are depicted in Figs. 1 and 2.

**Table 1:** The dosimetric comparison for the VMAT versus the IMPT plans

Organs	Dose levels	Lt. chest wall + regional nodes			Lt. chest wall			Rt. chest wall + regional nodes			Rt. chest wall		
		IMPT	VMAT	*P* value	IMPT	VMAT	*P* value	IMPT	VMAT	*P* value	IMPT	VMAT	*P* value
PTV	Dmax (GyRBE)	29.0 ± 1.0	28.0 ± 0.8	< 0.05	29.33 ± 1.4	28.1 ± 0.8	< 0.05	28.1 ± 0.8	27.4 ± 0.7	0.1	28.3 ± 1.1	26.4 ± 3.1	0.1
	D90 (GyRBE)	25.8 ± 0.3	25.6 ± 0.3	0.2	25.8 ± 0.6	25.4 ± 0.3	< 0.05	25.30 ± 0.51	25.4 ± 0.2	0.4	25.4 ± 0.5	25.3 ± 0.2	0.3
	D95 (GyRBE)	24.8 ± 0.1	24.8 ± 0.4	0.9	24.8 ± 0.2	24.7 ± 0.2	0.2	24.6 ± 0.2	24.7 ± 0.3	0.4	24.8 ± 0.5	24.9 ± 0.8	0.6
Ipsi-lung	Dmean (GyRBE)	8.3 ± 1.3	7.9 ± 1.7	0.4	7.4 ± 1.9	7.8 ± 0.8	0.5	7.3 ± 1.2	7.9 ± 2.7	0.5	6.9 ± 1.7	8.0 ± 1.2	0.1
	V5 (%)	55.1 ± 9.2	54.6 ± 11.9	0.9	49.3 ± 10.5	54.2 ± 7.0	< 0.05	47.6 ± 8.1	57.1 ± 10.9	< 0.05	44.6 ± 10.5	52.7 ± 12.1	0.1
	V10 (%)	39.3 ± 7.2	27.3 ± 8.2	< 0.05	33.3 ± 7.7	26.5 ± 4.0	< 0.05	34.8 ± 9.4	31.9 ± 4.5	0.4	29.6 ± 5.4	27.9 ± 6.4	0.4
	V20(%)	9.7 ± 3.7	7.6 ± 4.7	< 0.05	8.5 ± 5.9	6.4 ± 3.0	0.2	9.6 ± 3.4	8.2 ± 3.6	0.1	9.6 ± 4.3	8.3 ± 3.9	0.3
Contra-lung	Dmean (GyRBE)	0.2 (0.1–0.3)	3.4 (2.9–3.7)	< 0.05	0.1 (0.1–0.2)	3.1 (2.7–4.0)	< 0.05	0.1 (0.0–0.1)	3.1 (2.9–3.2)	< 0.05	0.0 (0.0–0.1)	2.6 (2.4–3.0)	< 0.05
	V5 (%)	0.6 (0.2–1.2)	20.9 (14.5–25)	< 0.05	0.4 (0–0.9)	14.1(11.1–26.8)	< 0.05	0 (0–0)	15.8 (12.4–18.7)	< 0.05	0 (0–0)	10.9 (9.4–15)	< 0.05
	V10 (%)	0.1 (0–0.3)	0.9 (0.6–2.1)	< 0.05	0 (0–0)	0.8 (0.4–1.5)	< 0.05	0 (0–0)	0 (0–0)	< 0.05	0 (0–0)	0.4 (0.2–0.6)	< 0.05
Heart	Dmax (GyRBE)	16.9 ± 7.0	19.9 ± 4.2	< 0.05	16.9 (10.4–25.5)	18.6 (18.3–22.0)	0.3	10.6 ± 4.8	10.1 ± 4.8	0.7	8.4 (4.8–13.4)	11.2 (8.4–14.5)	0.4
	Dmean (GyRBE)	1.1 (0.6–2.0)	4.6 (4.4–4.7)	0.1	1.2(0.7–1.5)	5 (4.6–5.6)	< 0.05	0.4 (0.3–0.7)	2.8 (2.2–3.4)	< 0.05	0.5 (0.2–0.8)	3.3 (2.5–3.5)	< 0.05
	V5 (%)	7.5 ± 4.0	28.2 ± 10.9	< 0.05	8.6 (4.6–10.2)	33 (26.3–40.2)	< 0.05	2.3 (1.6–4.7)	10.6 (5.4–17.1)	< 0.05	2.3 (0.5–5.6)	16.3 (6–17.8)	< 0.05
	V10 (%)	3.5 (1–6.3)	8.5 (7.4–11.8)	< 0.05	4 (1.1–5.2)	12 (8.3–14.4)	< 0.05	1.2 (0.2–2.2)	1.7 (0–3)	0.64	0.2 (0–1.2)	1.1 (0–2.5)	0.4
	V20 (%)	0.4 (0–0.8)	0.5 (0.4–1.7)	0.1	0.3 (0–0.6)	0.5 (0.2–0.6)	0.2	0 (0–0)	0 (0–0)	0.6	0 (0–0.1)	0 (0–0)	0.1
Skin	Dmax (GyRBE)	28.4 ± 1.2	27.3 ± 0.9	0.1	28.4 ± 1.9	27.3 ± 0.8	< 0.05	28.2 ± 1.1	26.8 ± 0.6	< 0.05	28.2 ± 1.4	26.8 ± 0.6	< 0.05
	Dmean (GyRBE)	24.5 ± 1.1	23.4 ± 1.0	< 0.05	24.7 ± 1.7	23.2 ± 0.9	< 0.05	25.2 ± 1.7	23.3 ± 0.7	< 0.05	24.9 ± 0.8	23.3 ± 0.6	< 0.05
Esophagus	Dmax (GyRBE)	24.2 ± 2.3	22.0 ± 4.2	< 0.05	2.0 (0.6–3.8)	8.7(8.3–9.6)	< 0.05	19.8 ± 4.3	19.4 ± 4.3	0.7	0.2 (0.1–2.4)	7.9 (5.6–9.8)	< 0.05
	Dmean (GyRBE)	4.5 ± 1.7	7.0 ± 1.7	< 0.05	0.2 (0.1–0.4)	3.4 (2.8–3.5)	< 0.05	2.9 ± 1.2	5. 7 ± 0.9	< 0.05	0 (0–0.2)	2.9 (2.5–3.0)	< 0.05
Thyroid	Dmax (GyRBE)	27.7 ± 1.1	27.1 ± 1.0	0.1	1.8(1.2–2.8)	0.8 (0.7–1)	0.0	26.1 ± 2.0	25.2 ± 3.9	0.4	0.6 (0.0–1.6)	0.8 (0.7–1.0)	0.9
	Dmean (GyRBE)	13.5 ± 6.0	16.3 ± 4.5	0.4	0.2 (0.1–0.3)	0.5 (0.4–0.6)	< 0.05	12.5 ± 2.7	14.8 ± 1.9	< 0.05	0.1 (0–0.3)	0.5 (0.4–0.6)	< 0.05

Mean ± SD for normally distributed data using a paired t-test; median (IQR) for nonnormally distributed data using the Wilcoxon signed-rank test

**Fig. 3:**
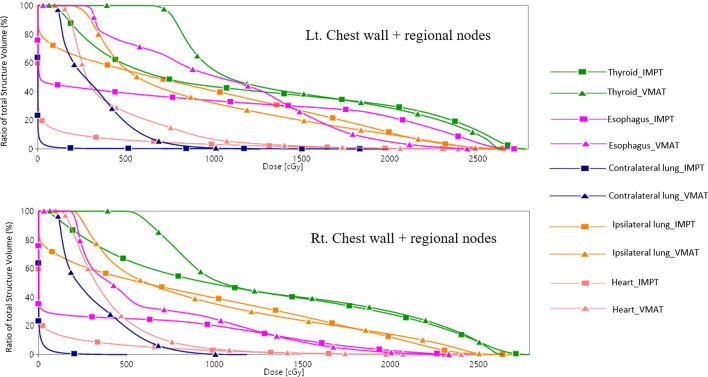
Dose-volume histograms for OARs of the VMAT and IMPT plans of the left (upper) and right (lower) chest walls with regional node irradiation

**Fig. 4:**
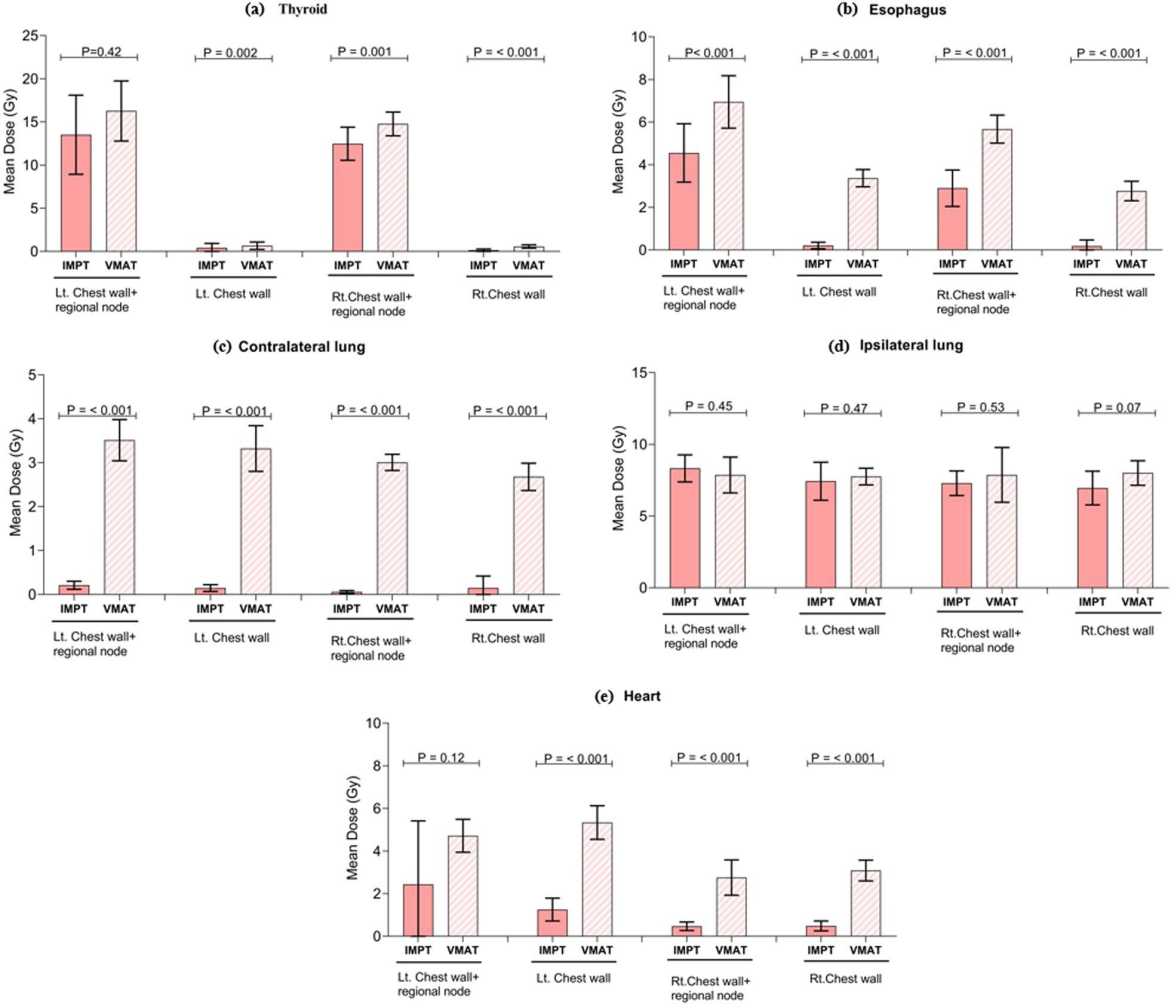
The mean dose of OARs at 95% CI: **a** thyroid; **b** esophagus; **c** contralateral lung; **d** ipsilateral lung; **e** heart

The ipsilateral lung mean dose of the IMPT plans was comparable to that of the VMAT plans. The ipsilateral lung dose was approximately 6.9–8.3 Gy(RBE) in IMPT and 7.8–8.0 Gy(RBE) in VMAT, which was not significantly different. While the V10 and V20 of IMPT were higher than those of VMAT. The V5 of the ipsilateral lung in IMPT was lower than that of VMAT in the left chest wall, the right chest wall, and the right chest wall with regional node plans. The advantage of IMPT over VMAT was observed in the contralateral lung and heart. A mean contralateral lung dose less than 1 Gy(RBE) was achieved by IMPT. The Dmean and V5 of the heart in the IMPT plans were approximately one-fourth of those in the VMAT plans. For the right-sided scenarios, the Dmean of the heart was below 1 Gy(RBE) in all scenarios of IMPT while it was 2.8–3.3 Gy(RBE) in the VMAT plans. IMPT gave a significantly higher skin dose due to the absence of a skin-sparing effect in the proton beam. The left chest wall plans demonstrated a higher esophageal dose than the right chest wall. The IMPT plans provided a significantly lower esophageal Dmean than the VMAT plans. The thyroid Dmean in the IMPT plans was less than that in the VMAT plans. A sample dose volume histogram (DVH) comparison of the left and right chest wall including regional nodes for the VMAT versus the IMPT plans is displayed in Fig. 3. The trends of DVHs of OARs were similar on both sides of the chest wall with regional node plans. The IMPT plan gave a better low-dose volume of the ipsilateral lung than the VMAT plan. The contralateral lung and heart were safe from the dose bath with the IMPT technique. The mean dose of OARs was also plotted with 95% CI and depicted in Fig. 4.

## Discussion

With low α/β, breast cancer is one of few malignancies for which a high dose per fraction provides a benefit in killing cancer cells [21]. Hypofractionation is the current standard in adjuvant radiotherapy and has been increasingly used in many centers. A new ultrahypofractionation regimen has recently been introduced and has demonstrated noninferiority in terms of acute skin toxicity and oncological outcomes when compared to standard hypofractionation [14]. Brunt et al. [12] published results of the FAST-Forward trial and concluded that ultrahypofractionation, 26 Gy in 5 fractions for 1 week, did not show a significant difference in terms of patient-assessed normal tissue effects, clinician-assessed normal tissue effects, photographic changes in breast appearance, and oncological outcomes compared with 40 Gy in 15 fractions. With this short radiotherapy treatment course, this dose-fractionation regimen could be an option during the COVID-19 pandemic. Piras et al. [22] reported a dosimetric study of the FAST-Forward protocol of post-conservative surgery left breast cancer using the VMAT technique compared with the 3D-CRT technique, while our study compared VMAT with IMPT in postmastectomy plans. Piras’s results showed an ipsilateral lung V30 of 8.33 Gy, while our study revealed V20 approximately 6.4–7.6 Gy in VMAT plans. Our center aims to introduce ultrahypofractionation using proton beams; thus, dosimetric comparison of the target volume and OARs with VMAT is necessary. Although the DVH of most OARs except the ipsilateral lung in IMPT is clearly superior compared with those of VMAT in our study, we cannot assert that proton therapy will become the standard treatment in breast cancer in the near future. Further clinical studies are needed to assess whether the dosimetric advantage will translate into a clinical benefit. In addition, cost effectiveness should be considered. The cost of radiotherapy sessions and the cost of treatment for related toxicities must be weighed. However, we believe that in some special cases in which cardiac sparing is needed, proton therapy could be an appropriate alternative treatment [23].

Regarding toxicities, chest wall irradiation carries the risk of radiation pneumonitis, cardiac mortality, and secondary cancers [15, 24, 25]. Our study evaluated the dosimetric parameters of VMAT and IMPT for ultrahypofractionation postmastectomy in breast cancer. The PTV coverage was equivalent for IMPT versus VMAT plans. These results corresponded well with Ares’s study [6] which showed IMPT reduced the bath of low dose distribution for OARs. Our study demonstrated that the V5 and Dmean of the ipsilateral lung in IMPT were less than those in VMAT in 3 out of 4 scenarios. In lung cancer patients, the large volumes of lung that receive low-dose irradiation (5 Gy or 10 Gy) increase the rate of pneumonitis [26]. The volume of lung tissue receiving radiation could be associated with the risk of late toxicity, including second malignancy, particularly in young women. Our study confirmed that both high-dose and low-dose exposure to normal tissues were less common in the IMPT plan, which corresponded with MacDonald’s report [24].

Heart dose is well known to be correlated with cardiac morbidity [27]. Our study supported that IMPT can reduce the potential risk to the cardiac structure. MacDonald’s study [24] reported that proton therapy allows for the treatment of deep-seated lymph nodes, such as the internal mammary lymph node (IMN), with minimal cardiopulmonary doses. Our results also support that V5, V10 and the mean heart dose were much lower in IMPT than in VMAT. With this dosimetric advantage, lower long-term morbidity from heart disease could be expected from proton beam therapy.

One concern with IMPT to the chest wall from this study is the increased dose to the skin because of the lack of a skin-sparing effect with protons. However, the total dose of our regimen was only 26 Gy(RBE), and the actual differences in doses between IMPT and VMAT are negligible. Therefore, acute skin toxicity, which typically relates to the total radiation dose, should not be greatly affected. We predict that this dose level is feasible and will be well-tolerated by patients. However, cosmesis is also an important outcome. Data collection in clinical studies is in progress and is reported in the near future.

Only 3 mm and 3.5% robust CTV optimization was sufficient and applied in this study because breathing motion management was concerned with access in clinical use. In addition, chest wall volumes are usually superficial in depth, ranging from 3 cm or less, and there was very little uncertainty about intrafraction motion for postmastectomy patients [23, 26]. Depauw et al. [28] reported that the patient’s chest wall movement along the AP/longitudinal direction was approximately 3 mm when motion management was performed during treatment. In addition, the beam path in proton plans is parallel to the target movement direction, which results in a minimal change in the position of the target; thus, the overall dosimetric impact is below 1% [6, 29].

Even though our study provided insights into proton and photon therapy in ultrahypofractionated postmastectomy irradiation, there are some limitations. First, only ten plans were evaluated for each scenario, and a large sample would provide more reliable data. Second, this is a dosimetric study, and the clinical outcomes of the proton interventions are needed. Nonetheless, the dose constraints to PTV and OARs of this study have been applied in our clinical practice, and we plan to report the clinical outcomes in the future. Additionally, this study does not compare the effectiveness of TCP and NTCP for both techniques. However, because this study's dose comparison in OARs was based on an equivalent prescription dose, the physical dose comparison may be rational. Clinical outcomes pertinent to the TCP and NTCP will be reported in the future.

## Conclusion

IMPT showed better normal tissue sparing while maintaining PTV coverage than VMAT in postmastectomy irradiation using ultrahypofractionation. Despite higher the dose to the skin in the IMPT group, the actual difference was negligible. IMPT could significantly reduce the dose to adjacent organs at risk, which could translate into reduced late toxicities compared with those of the photon plan. Further clinical studies are needed to prove the feasibility of this dose fractionation regimen using proton beam therapy. Our proposed dosing scheme would not strongly affect the acute toxicities and would be more convenient to use in COVID-19 pandemic situations.

## Data Availability

All data generated or analyses during this work are included in this published article.

## Abbreviations

CT: Computed tomography
CTV: Clinical target volume
3D-CRT: Three-dimensional conformal radiotherapy
DVHs: Dose volume histograms
IMPT: Intensity modulated proton radiation therapy
IMRT: Intensity modulation radiotherapy
PBT: Proton beam therapy
PTV: Planning target volume
VMAT: Volumetric arc radiation therapy
OARs: Organs at risk
